# Predictors of Residual Right to Left Shunt in Patients Undergoing Percutaneous Transcatheter Patent Foramen Ovale Closure: A New Clue “Inferior Vena Cava-Patent Foramen Ovale Angle”

**DOI:** 10.3390/jcm13226703

**Published:** 2024-11-07

**Authors:** Duygu Inan, Benay Ozbay, Ayse Irem Demırtola Mammadli, Funda Ozlem Pamuk, Sevil Tugrul Yavuz, Emir Derviş, Yeliz Guler, Duygu Genç Albayrak, Kadir Kasım Sahin, Alev Kılıcgedik

**Affiliations:** 1 Department of Cardiology, Basaksehir Cam and Sakura City Hospital, 34480 Istanbul, Türkiye; kof.12@hotmail.com (F.O.P.); seviltugrul88@gmail.com (S.T.Y.); yelizguler829@gmail.com (Y.G.); drduygugenc@gmail.com (D.G.A.); kadirsahin944@gmail.com (K.K.S.); akilicgedik@yahoo.com (A.K.); 2 Department of Cardiology, Heart and Vascular Institute, University of Pittsburg Medical Centre, Pittsburg, PA 15213, USA; benayozbay@hotmail.com; 3 Department of Cardiology, Ankara Bilkent City Hospital, 06800 Ankara, Türkiye; airem90@gmail.com; 4 Department of Cardiology, Medipol University, Bahcelievler Hospital, 34180 Istanbul, Türkiye; emirdervis@hotmail.com

**Keywords:** patent foramen ovale, residual shunt, structural features

## Abstract

**Background:** Many different devices are currently used for percutaneous patent foramen ovale (PFO) closure (pPFOc), aiming to achieve complete occlusion to prevent recurrent embolism. We aimed to reveal the association between residual right-to-left shunt (RLS) after pPFOc and baseline structural features of the PFO determined using transoesophageal echocardiography (TEE) before the procedure. **Methods:** This is a single-center cross-sectional study. A total of 123 patients who underwent pPFOc for cryptogenic stroke (CS) were screened, consecutively. Patients were compared based on the presence of residual RLS. The association of structural features of the PFO with residual RLS was evaluated using logistic regression analysis. **Results:** The procedure was successfully completed in a total of 103 patients included in the study after exclusion and 21% had significant residual RLS. During a median follow-up of 18 months, one patient died at 25 months due to malignancy, recurrent CS were observed in 5 (5%) patients, and atrial fibrillation was detected in 3 (3%) patients. No significant difference was observed in the baseline clinical characteristics and laboratory parameters among the patients. In addition to atrial septal aneurysm (ASA), aortic rim, tunnel width and length; inferior vena cava (IVC)-PFO tunnel angle was associated with residual RLS with a cut-off 11.3 (AUC: 0.786, 64% sensitivity, 87% specificity, *p* < 0.001). In individuals with residual RLS, longer and wider tunnel size, rudimentary aortic rim, acute IVC-PFO tunnel angle, and decreased tunnel length-left disc ratio were observed. **Conclusions:** IVC-PFO tunnel angle is a novel parameter and provides benefit to detect significant RLS in pPFOc patients.

## 1. Introduction

Patent foramen ovale (PFO) is an anatomical connection between the right and left atrium, found in approximately 25–30% of the population, and serves as a pathway for a paradoxical embolism (PE) [1]. It is associated with various conditions such as cryptogenic stroke (CS), transient ischemic attack (TIA), peripheral emboli, decompression sickness, and migraine [2,3,4,5]. PFO is present in about half of CS patients, often accompanied by several structural features such as atrial septal aneurysm (ASA) and large tunnel width (≥2 mm) [2,5,6]. Multiple randomized studies and meta-analyses have shown that percutaneous PFO closure (pPFOc) reduces recurrent events as a secondary prevention for patients with PE and CS [7,8,9,10]. pPFOc is the treatment method recommended in current guidelines to prevent recurrent stroke in patients with PFO-related stroke [5]. Various occluder devices are used in pPFOc, along with alternative methods such as percutaneous suture-mediated PFO closure or surgical PFO closure [5,7,8,9,10,11,12]. Despite a high procedural success rate, residual right-to-left shunt (RLS) is observed in approximately one-quarter of patients [5,13,14]. This is significant because recurrent paradoxical embolic events are observed in patients with residual RLS, highlighting the importance of achieving complete closure with all pPFOc devices and methods [5,9,10,11,12,13,14].

The rate of residual RLS after pPFOc varies depending on the type of device used, closure method, and duration of follow-up period, but it is known that certain anatomical features of the PFO are key determinants [15]. ASA, large tunnel width, and small aortic rim, which are morphological risk markers of PFO in terms of embolism, are parameters that have been shown to be associated with residual RLS [15,16]. A review of the literature reveals a limited number of studies examining the relationship between the morphological features of PFO and residual RLS, indicating a need for more dedicated research on these morphological characteristics. For this purpose, we evaluated the association between residual RLS after pPFOc and baseline structural features of the PFO determined using transoesophageal echocardiography (TEE) before the procedure.

## 2. Methods

### 2.1. Study Design and Study Participants

Between May 2020 and 2023, a total of 123 consecutive patients younger than 60 years old who underwent pPFOc due to CS (ischemic stroke or recurrent TIA) were screened, consecutively. All patients had extensive neurological and cardiological evaluations, including transthoracic echocardiography (TTE) or microbubble test with TTE/TEE, brain computed tomography or magnetic resonance imaging, 24 or 48 h Holter monitoring, Doppler ultrasound of supra-aortic vessels, and inherited or acquired thrombophilia. When all these tests were evaluated together, the entire study population met the current indications for pPFOc [5]. In our center, residual RLS is routinely evaluated by control microbubble test with TTE at 1, 6, and 12 months post-pPFOc. TEE is also performed in cases with residual shunt or suspected bubble study. The microbubble test was performed at rest and during maximal valsalva with approximately 5–8 cc of agitated saline. Additionally, all pPFOc procedures are performed under TEE guidance. In the conduct of the study, patient cards in the hospital registration system or telephone interviews were utilized for the collection of demographic, laboratory and clinical information, and follow-up findings. A total of 20 patients were excluded from the study who did not have pre-procedural TEE images, had inadequate images for structural measurements, had cribriform septum primum or concomitant secundum atrial septal defect, required treatment with open heart surgery for other reasons, did not have follow-up evaluations with bubble tests, and whose follow-up and baseline characteristic data could not be obtained through hospital medical records or telephone interviews. This study was conducted with the approval of the institutional ethical committee and in accordance with the ‘Good Clinical Practices’ guidelines of the Helsinki Declaration.

### 2.2. Definitions and Echocardiographic Evaluation

**Figure 1:**
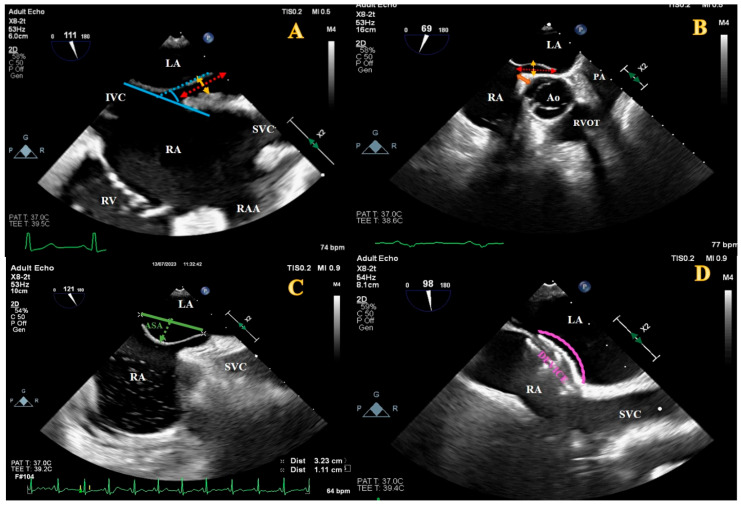
Illustration of some structural features of PFO on transoesophageal echocardiography. (**A**) Pre-procedure bi-caval imaging. Tunnel length, red dashed arrow; Tunnel width, yellow dashed arrow; IVC-PFO tunnel angle, blue dashed area. (**B**) Mid-oesophageal short axis imaging. Orange thick arrow, aortic rim. (**C**) Pre-procedure bi-caval imaging of ASA. ASA, green dashed area. (**D**) Post-procedure bi-caval imaging. Device left disc size, pink sawed line. Ao, aortic valve; ASA, atrial septal aneurysm; IVC, inferior vena cava; LA, left atrium; PA, pulmonary artery; PFO, patent foramen ovale; RA, right atrium; RAA, right atrial appendix; RV, right ventricle; RVOT, right ventricular outflow tract; SVC, superior vena cava.

Stroke and TIA were defined as previously described in guidelines [17]. TEE images of all patients before and during the pPFOc procedure were evaluated by two independent imaging specialists blinded to clinical information and follow-up data. In TEE images of all patients, basic structural features were systematically evaluated as recommended in the guidelines [5,16]. PFO tunnel length and width (maximum overlap and gap distance of the septum primum and septum secundum, respectively), presence and degree of spontaneous/provoked RLS, ASA (atrial septum ≥ 15 mm base diameter with ≥10 mm excursion), vena cava inferior (VCI)-PFO tunnel angle, aortic rim, PFO tunnel length device/left disc ratio, lipomatous hypertrophy, embryonic or fetal remnants such as Chiari’s network, and Eustachian valve were identified (Figure 1). An inter atrial septum thickness ≥ 15 mm on TEE is considered lipomatous hypertrophy, while a prominent Eustachian valve is defined as having a length of more than 10 mm [16]. The angle between the IVC and the PFO flap was measured in an imaging plane (between 30–120 degrees at the mid-eosophageal level) that displayed the IVC and interatrial septum [18,19]. Residual RLS was evaluated by a cardiologist blinded to the post-procedural TTE images through the hospital record system. TTE images recorded by injecting agitated saline at rest and during valsalva were analyzed. Residual shunt was classified as follows: negative shunt, no microbubble; mild shunt, 1–5 microbubbles; moderate shunt, 6–20 microbubbles; and severe shunt, >20 microbubbles observed [12,16]. At 12 months, patients with moderate and severe shunt were considered to have residual RLS. TTE and TEE procedures were performed with the EPIQ CVx cardiovascular ultrasound system and X5-1 and X8-2t transducers (PHILIPS, Cambridge, MA, USA). Post-hoc TTE and TEE analyses were conducted using cardiovascular ultrasound measurement software (QLAB 10).

### 2.3. Percutaneous PFO Closure Procedure

Percutaneous PFO closure was performed by experienced operators under fluoroscopy and TEE guidance with sedation. In all patients, the procedure was performed through the femoral vein to pass through the PFO. No balloon sizing or septal puncture was performed in any patients. Based on TEE measurements before and during the procedure, the appropriate size AMPLATZER or OCCLUTECH devices were implanted. The implanted devices were classified into three groups for the analysis of the study. Group I (medium size devices) comprised 23/25 mm OCCLUTECH devices, group II (small devices) comprised 18 × 18 mm AMPLATZER devices, and group III (large devices) comprised 27 × 30 mm OCCLUTECH and 30 × 30 AMPLATZER devices. Large devices were implanted in patients with more complex anatomy with prominent ASA, thick septum secundum, and tunnel length > 15. Small devices were implanted in patients with short PFO tunnel and immobile septum primum. All patients were prescribed acetylsalicylic acid 100 mg and clopidogrel 75 mg before the procedure and treatment was continued for 6 months. After 6 months of dual antiplatelet therapy, 6 patients with recurrent stroke or atrial fibrillation at follow-up were switched to anticoagulant therapy, while the other patients were treated with acetylsalicylic acid 100 mg alone.

### 2.4. Study Outcome

Main outcome was considered residual RLS at 12-month follow-up. Mortality, recurrent cryptogenic stroke, and atrial fibrillation during follow-up were also evaluated.

### 2.5. Statistical Analysis

Standard statistical software programs were used (SPSS, version 29, SPSS Inc., Chicago, IL, USA and R Project for Statistical Computing, version 3.5.3, Vienna, Austria). Continuous variables are expressed as mean (±standard deviation) and median (±interquartile range), and categorical variables are expressed as percentages. The Student’s *t*-test was used to compare normally distributed continuous variables between independent groups, while the Mann-Whitney U test was used for non-normally distributed variables. Diagnostic accuracy was assessed using receiver operating characteristic (ROC) curves, with cut-off points determined using the Youden index [20]. Areas under the ROC curves (AUCs) were compared using the method of DeLong et al. [21]. A multivariate logistic regression model identified independent determinants of residual RLS in the study population. Intraclass correlation coefficients (CC) were calculated to assess intra- and inter-observer variations for the tunnel length/device to left disc ratio and the VCI-PFO tunnel angle, based on 10 random studies. Repeatability measurement for the IVC/PFO angle was performed over one video loop. ASA was monitored in one of these 10 studies. These parameters are less well established for clinical use compared to the aortic rim and tunnel width. A two-sided *p*-value of <0.05 was considered statistically significant.

## 3. Results

### 3.1. Patient Characterıstics and Clinical Follow-Up

A total of 123 patients were screened, and 103 who did not meet the exclusion criteria were included in this study. The pPFOc procedure was successfully completed in all patients without any procedure-related complications. At 12 months after pPFOc, 22 (21%) patients had residual RLS. During a median follow-up of 18 months (IQR: 14–26 months, range: 12–46 months), a patient died at 25 months due to metastatic testicular tumor, recurrent CS were observed in 5 (5%) patients, and atrial fibrillation was detected in 3 (3%) patients. Only 2 patients had one fenestration immediately adjacent to the PFO tunnel. No residual RLS was observed in these two patients after pPFOc with a group II device. Tunnel length/device left disc ratio was significantly decreased in patients with residual RLS compared to those without (*p* = 0.022). Tunnel length was significantly longer in patients with residual RLS compared to those without (*p* = 0.009). Tunnel width was significantly larger in patients with residual RLS compared to those without (*p* < 0.001). The aortic rim was more rudimentary in patients with RLS than in those without (*p* < 0.001). The VCI-PFO tunnel angle was more acute in patients with residual RLS than in those without (*p* < 0.001). Prominent ASA was more prevalent in patients with residual RLS (*p* < 0.001). No significant difference was observed in other demographic, clinical, laboratory, and structural PFO parameters (Table 1 and Table 2). When the devices used in pPFOc procedure were analyzed, the most commonly implanted device was 23/25 mm OCCLUTECH (48%), followed by 18 × 18 mm AMPLATZER (40%).

### 3.2. Structural Features of Residual RLS

**Figure 2:**
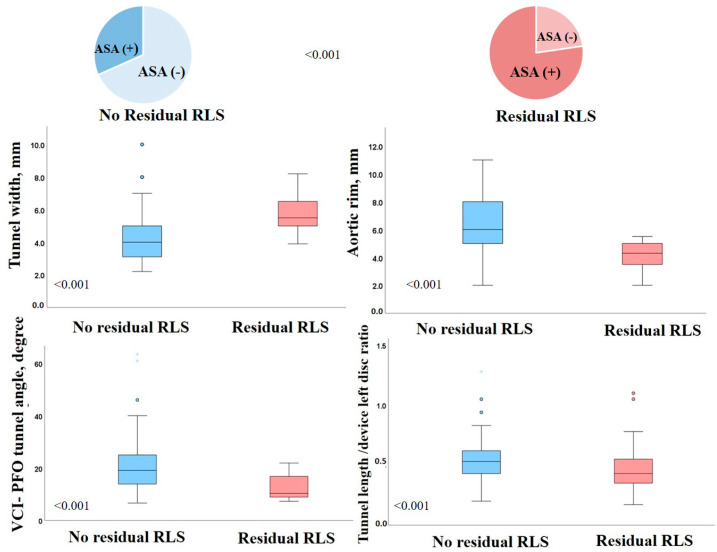
Box plot and pie chart of the presence and absence of residual shunt according to different structural features of PFO. ASA, atrial septal aneurysm; RLS, residual right-to-left shunt.

**Figure 3:**
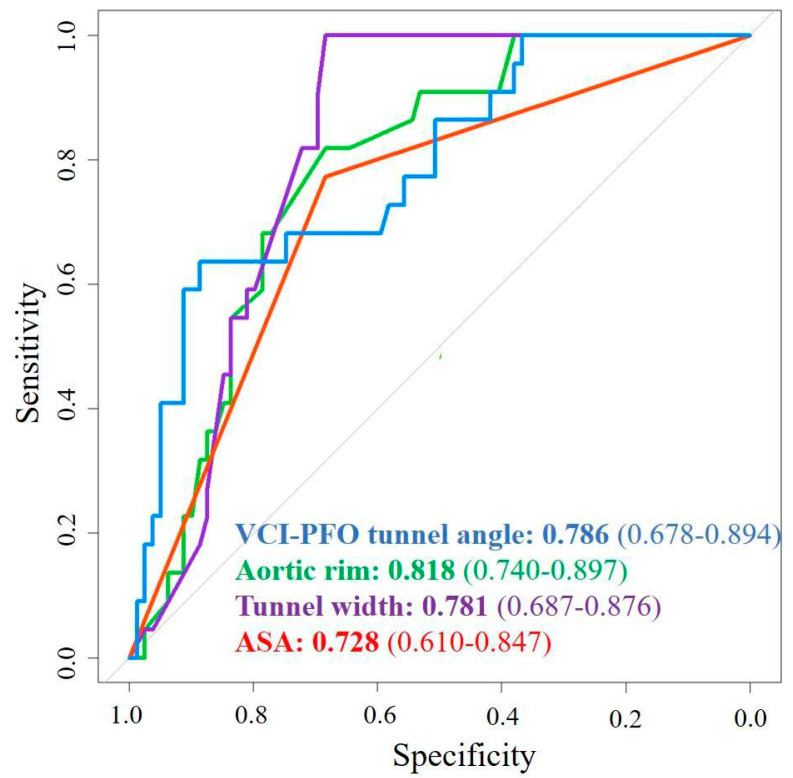
Comparison of associations of structural parameters with residual RLS. ASA, atrial septal aneurysm; PFO, patent foramen ovale; RLS, right to left shunt; VCI, vena cava inferior.

**Figure 4:**
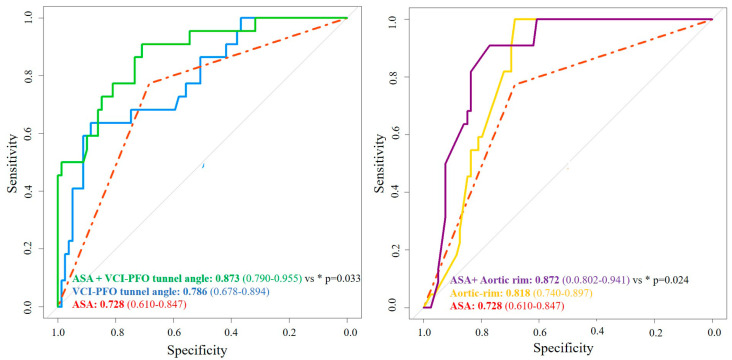
ROC analysis of ASA, aortic rim, and IVC-PFO tunnel angle. ASA, atrial septal aneurysm; AUC, area under curve; CI, confidence interval; PFO, patent foramen ovale; ROC, receiver operating characteristic; RLS, right to left shunt; VCI, vena cava inferior. * Represents Delong *p* vs. ASA.

Tunnel width, aortic rim, ASA, and VCI-PFO tunnel angle were significant predictors of residual RLS in univariate regression analysis. Tunnel width (OR: 2.17, 95% CI: 1.30–3.63, *p* = 0.003), aortic rim (OR: 0.47, 95% CI: 0.28–0.79, *p* = 0.004), ASA (OR: 8.45, 95% CI: 1.88–38.05, *p* = 0.005), and VCI-PFO tunnel angle (OR: 0.80, 95% CI: 0.69–0.93, *p* = 0.003) also remained significant predictors in multivariate regression analysis (Table 3). The association between each conventional echo parameter and significant residual RLS was illustrated using box plots and pie charts (Figure 2). Furthermore, in the ROC curves analysis, the correlation of residual shunt with conventional and new parameters was evaluated (Figure 3). Both the VCI-PFO angle and the aortic rim added incremental value to the ASA (Figure 4). In addition to the aortic rim, the IVC-PFO tunnel angle predicted residual RLS with a cut-off of 11.3 (AUC: 0.786, 64% sensitivity, 87% specificity, *p* < 0.001) (Figure 4). The intra-observer and inter-observer correlation coefficients for tunnel length device/left disc (intraclass CC: 0.999, 95% CI: 0.996–1.000 and interclass CC: 0.998, 95% CI: 0.994–1.000) and VCI-PFO tunnel angle (intraclass CC: 0.999, 95% CI: 0.998–1.000 and interclass CC: 0.993, 95% CI: 0.977–0.998) were all very high, indicating excellent measurement reliability.

## 4. Discussion

The main findings of this study were as follows: (i) Residual RLS was observed in 21% of patients at approximately 12 months after pPFOc procedure; (ii) IVC-PFO tunnel angle was a novel and reliable parameter associated with residual RLS after pPFOc procedure; (iii) ASA, tunnel width, and aortic rim were other important parameters associated with residual RLS; (iv) no complications were observed in our pPFOc procedures performed under TEE and fluoroscopy guidance.

Despite high procedural success rates of pPFOc, it has been reported that a significant number of patients still experience residual RLS, which poses a risk for recurrent ischemic events. The rate of residual RLS varies according to the definition of RLS, type of device, echocardiographic techniques, and follow-up time, and comparison of results is difficult [13,14,22]. In different studies, it has been shown that RLS can be observed at rates of up to approximately 25–34% [14,22,23,24,25]. Marchese et al. [23] reported that 27 (21.6%) and 17 (13.6%) patients had significant residual RLS after implantation of the Amplatzer device at 3 and 12 months, respectively. In another study published by Deng et al. [24] in which different devices were implanted with a mean follow-up of 3.7 years, this rate was 22%. Furthermore, in this study, the presence of a residual shunt, especially a medium or large residual shunt, was associated with an increased risk for stroke or TIA recurrence [24]. In our study, residual RLS was observed in 21% of patients at 12 months after pPFOc procedure. This rate was similar to the literature. Although recurrent stroke was detected in 5 (5%) patients in our study, contrary to some literature [22,23,24], the event rate was similar between the groups with and without residual RLS. The relatively short median follow-up time and the inclusion of patients with 5 to 10 bubble shunts in the RLS group, who are known to be at lower stroke risk, may explain the finding of these results.

Different structural features such as spontaneous RLS, prominent ASA, wide and long tunnel, prominent Chiari’s network and Eustachian valve, rudimentary aortic rim, and acute inferior vena cava angle are known to be associated with systemic embolism in PFO patients [6,13,18]. The presence of ASA and tunnel length have been reported as the most proven parameters associated with residual RLS in patients undergoing PFO closure [18,22,23,24,25,26]. The presence of ASA increases the complexity of the PFO structure which may prevent complete closure [24,26]. Similarly, a larger tunnel size makes it difficult for closure devices to achieve complete sealing [23,24]. The aortic rim, a parameter well known from atrial septal defect closure patients and associated with long-term aortic erosion, is also important in PFO closure patients [27]. A rudimentary or underdeveloped aortic rim limits the anchoring and sealing capacity of the closure device in these patients [5,16,27]. In our study, the prevalence of ASA was significantly higher in patients with residual RLS, while PFO tunnels were significantly longer and wider. Moreover, a rudimentary aortic rim was more common in patients with residual RLS. An acute IVC-PFO tunnel angle was identified as a new and important predictor of residual RLS in this study. It is known that the angle between the IVC and PFO tunnel changes with age and decreases as age advances, contributing to the direct flow of blood from the IVC to the left atrium [26,28]. Indeed, an acute IVC-PFO tunnel angle is considered a feature of high-risk morphology for paradoxical embolism in individuals with PFO [18,28]. Similarly, in pPFOc patients, an acute angle can facilitate the direction of blood flow to the left atrium, hindering optimal device placement and manipulation, thereby contributing to the formation of residual shunts. Considering all these anatomical challenges, certain practices may help improve procedural success and reduce the risk of residual RLS. In patients with ASA, it is known that balloon sizing of the PFO tunnel reduces the risk of residual RLS [29,30]. Additionally, in patients with long and rigid tunnels, creating a controlled iatrogenic fenestration at the tunnel entrance, enhancing tunnel compliance using the balloon pull-back technique, or performing controlled balloon angioplasty to shorten and widen the tunnel can facilitate effective device implantation [30]. Moreover, devices designed with long and adaptable inter-atrial occluders that accommodate long tunnel morphology, as well as structures consisting of tunnel components without atrial occluders or suture-based non-device systems, may offer newer alternative methods for PFO closure in patients with complex morphologies [12,15,31]. However, the primary challenge with these options is the limited experience and insufficient data. Since the IVC-PFO tunnel angle was found to be a new high risk criterion for residual shunting after PFO closure procedure, it may be taken into consideration for the device type, device size, or selection of these newer methods.

In a study conducted by Vitarelli and colleagues [32] that examined patients with implanted PFO closure devices, it was shown that the occluder diameter varied according to the size of the atrial septum and the PFO size assessed by pre-implantation TEE examination. Additionally, it was emphasized that in patients with septal aneurysm and complex PFOs the ratio of device size to PFO diameter significantly increased compared to those with simple PFO morphology (mean, 3.7:1 vs. 2.5:1, *p* < 0.05). In our study, we also evaluated the ratio of PFO tunnel length to the device left disc. We speculated that the mismatch in this ratio, particularly in patients who had smaller devices due to longer PFO tunnels and smaller total septum lengths and atrial diameters, might affect residual shunting. Although this was not found to be statistically significant in regression analysis, this ratio was lower in the residual RLS group. It has been previously reported that septal puncture was not used during the pPFOc procedure in our study population. Based on these findings, we thought that in patients needing a larger device and with a low PFO tunnel length/device left disc ratio, particularly in long and narrow tunnels, septal puncture could be more effective for procedural success than attempting to navigate through the PFO tunnel. Considering the relatively small size of our study population, we anticipate that this conclusion would yield more valuable results when evaluated in a larger group. It can be stated that in this study, residual RLS demonstrated a stronger association with the complexity of PFO morphology compared to the potential influence of device type, device size, or technique.

The imaging modality preference during pPFOc plays a critical role in the safety and efficacy of the procedure [16,30]. In this study, no complications were observed in pPFOc procedures performed under TEE and fluoroscopy guidance. This result was consistent with the findings in the literature and supported the safety and efficacy of the combination of TEE and fluoroscopy [16,30,33,34]. The fact that TEE provides high-resolution imaging and fluoroscopy offers instantaneous guidance provided safe guidance at every stage of the procedure. Thus, in addition to reducing complication rates, significant advantages in improving device placement accuracy have been demonstrated [30,33,34,35]. Intracardiac echocardiography (ICE), a newer imaging modality, is a valuable alternative to TEE for guiding pPFOc [15,16]. Performed by interventional cardiologists, ICE uses a catheter to provide high-resolution images that help assess the size and position of the PFO and guide device deployment [15,30]. It also allows for immediate detection of residual shunting and monitoring of potential complications while minimizing the need for general anesthesia and reducing radiation exposure [36]. However, its use is limited by costs, the need for specialized training, and potential complications related to femoral puncture [16,30].

### Study Limitations

This study has some limitations. Firstly, the sample size was relatively small and the findings were based on a single-center experience. The small sample size resulted in an insufficient number of results and led to difficulties in statistically validating the association between residual RLS and PFO structural features. Additionally, the exclusion of patients without pre-procedural TEE images or insufficient follow-up data may introduce selection bias. Larger multicenter studies are needed to validate these predictors and improve device selection algorithms. Secondly, the median follow-up period was 18 months, which may not cover all long-term complications or recurrent events. Extended follow-up studies are necessary to assess the durability of the closure and the long-term impact of residual RLS. We would like to raise concerns about measuring the IVC-PFO angle. Our literature review showed that angles were typically measured using mid-esophageal images at 30–120 degrees, where the IVC line often ran parallel to the septum primum. To obtain accurate measurements, we focused on the clearest images aligned with both the tunnel opening and the IVC. While evaluating patients, we extended a line along the septum primum, visualizing the IVC’s direction to ensure it remained parallel to the IVC, and drew another line parallel to the tunnel axis. However, any shifts in these lines may still introduce measurement errors. Although similar measurements between different observers may validate the reliability of the measurements, this represents another significant limitation of our study. Notwithstanding these limitations, the study provides valuable insights into the structural predictors of residual RLS following pPFOc and highlights the importance of comprehensive pre-procedural assessment using TEE. Future research should explore the role of newer imaging modalities and advanced closure devices that could address the structural challenges identified in this study.

## 5. Conclusions

This study highlights the importance of detailed anatomical evaluation using TEE in predicting residual RLS after pPFOc. The acute IVC-PFO tunnel angle, prominent ASA, tunnel width, and rudimentary aortic rim, among other structural features, have shown a strong association with residual RLS. IVC-PFO tunnel angle is a novel parameter and provides benefit to detect significant RLS in pPFOc patients. In patients exhibiting these characteristics, the choice of device type, size, and procedural management can be personalized. It is anticipated that the emergence of these findings will serve as a catalyst for future research and pave the way for new approaches in the management of PFO patients.

## Figures and Tables

**Table 1:** Baseline characteristics of the study population.

Variables	No Residual RLS (*n* = 81)	Residual RL (*n* = 22)	*p*-Value
Age, years	42.0 ± 9.5	40.0 ± 9.6	0.378
Male, n (%)	41 (51%)	11 (50%)	0.958
DM, n (%)	22 (27%)	3 (14%)	0.197
HT, n (%)	29 (36%)	6 (27%)	0.451
CAD, n (%)	12 (15%)	2 (9%)	0.452
Hyperlipidemia, n (%)	29 (36%)	7 (32%)	0.723
Prior ischemic stroke, n (%)	74 (91.1%)	20 (90.9%)	0.973
TIA, n (%)	7 (8.9%)	2 (9.1%)	0.973
Migraine, n (%)	6 (7.6%)	2 (9.1%)	0.818
DVT, n (%)	1 (1.3%)	1 (4.5%)	0.329
New onset AF, n (%)	3 (3.8%)	0 (0%)	0.353
Recurrent stroke, n (%)	3 (3.8%)	2 (9.1%)	0.311
RoPe score	6.9 ± 1.6	7.6 ± 1.4	0.076
Smoking, n (%)	28 (35%)	9 (41%)	0.615
Hemoglobin, g/dL	15.1 ± 1.6	13.1 ± 1.8	0.570
WBC, 10^9^/L	8.2 ± 2.4	8.5 ± 2.6	0.597
PLT, 10^9^/L	260 ± 88	268 ± 59	0.637
GFR, mL/min/1.73 m^2^	101 ± 19	108 ± 10	0.105
Fasting glucose, mg/dL	109 ± 32	94 ± 16	0.044
Total protein, g/L	68 ± 6.4	70 ± 4.4	0.267
Homocysteine, μmol/L	14.2 ± 8.8	11.6 ± 3.3	0.172
Total cholesterol, mg/dL	177 ± 42	173 ± 37	0.713
HDL, mg/dL	44 ± 11	43 ± 9	0.793
LDL, mg/dL	105 ± 35	107 ± 33	0.822
Triglyceride, mg/dL	151 ± 95	121 ± 41	0.144
PFO occluder device			0.932
Group I, n (%)	38 (46.9%)	11 (50%)	
Group II, n (%)	33 (40.7%)	8 (36.4%)	
Group III, n (%)	10 (12.3%)	3 (13.6%)	

AF, atrial fibrillation; CAD, coronary artery disease; DM, diabetes mellitus; DVT, deep vein thrombosis; GFR, glomerular filtration rate; HDL, high density lipoprotein; HT, hypertension; LDL, low density lipoprotein; PFO, patent foramen ovale; PLT, platelet; RLS, right to left shunt; TIA, transient ischemic attack; WBC, white blood cell.

**Table 2:** Echocardiographic features of the study population.

Variables	No Residual RLS (*n* = 81)	Residual RLS (*n* = 22)	*p*-Value
Tunnel length, mm	10.9 (9.0–13.0)	14.0 (9.6–16.2)	0.009
Tunnel width, mm	4.5 ± 1.6	5.7 ± 1.2	<0.001
Septum primum, mm	32.2 ± 6.4	30.0 ± 4.7	0.136
Septum secundum, mm	20.6 ± 5.1	19.9 ± 3.9	0.558
Aortic rim, mm	6.4 ± 2.1	4.3 ± 0.9	<0.001
Sinus valsalva diameter, mm	32.4 ± 4.7	31.1 ± 3.7	0.243
Ascendant aorta diameter, mm	30.7 ± 4.5	30.6 ± 4.4	0.956
VCI-PFO tunnel angle, degree	21.0 ± 10.6	12.5 ± 4.9	<0.001
Lipomatous hypertrophy, n (%)	7 (9%)	4 (18%)	0.215
Atrial septal aneurysm, n (%)	26 (32%)	17 (77%)	<0.001
Chiari network, n (%)	15 (19%)	2 (9%)	0.273
Eustachian valve, n (%)	19 (19%)	2 (9%)	0.774
Crista terminalis, n (%)	10 (12%)	2 (9%)	0.746
Spontaneous color Doppler shunt, n (%)	43 (53%)	9 (41%)	0.309
Bubble shunt at rest, n (%)	42 (52%)	11 (50%)	0.875
Bubble shunt with valsalva, n (%)	70 (87%)	21 (95%)	0.342
Tunnel length/device left disc ratio	0.53 (0.43–0.63)	0.43 (0.34–0.56)	0.022
Large PFO device (Group III) n (%)	10 (12.3%)	3 (13.6%)	0.872

PFO, patent foramen ovale; RLS, right to left shunt; VCI, vena cava inferior.

**Table 3:** Univariate and multivariate logistic regression analysis of structural features of PFO for prediction of residual RLS.

	Univariate Regression		Multivariate Regression	
	OR (95% CI)	*p*	OR (95% CI)	*p*
Tunnel width, mm	1.62 (1.19–2.20)	0.002	2.17 (1.30–3.63)	0.003
Aortic rim, mm	0.52 (0.37–0.73)	<0.001	0.47 (0.28–0.79)	0.004
Atrial septal aneurysm (Presence/Absence)	0.73 (2.44–22.2)	<0.001	8.45 (1.88–38.05)	0.005
VCI-PFO tunnel angle, degree	0.83 (0.75–0.93)	<0.001	0.8 (0.69–0.93)	0.003
Tunnel length/device left disc	0.15 (0.1–2.69)	0.195	-	-

CI, confidence interval; OR, odss ratio; PFO, patent foramen ovale; RLS, right to left shunt; VCI, vena cava inferior.

## Data Availability

The datasets used and/or analyzed during the current study are available from the corresponding author on reasonable request.
